# Effects of a behavioral intervention on physical activity, diet, and health-related quality of life in pregnant women with elevated weight: results of the HIPP randomized controlled trial

**DOI:** 10.1186/s12966-022-01387-w

**Published:** 2022-12-09

**Authors:** Sara Wilcox, Jihong Liu, Gabrielle M. Turner-McGrievy, Alycia K. Boutté, Ellen Wingard

**Affiliations:** 1 Prevention Research Center, Arnold School of Public Health, University of South Carolina, 921 Assembly Street, Columbia, SC 29208 USA; 2 Department of Exercise Science, Arnold School of Public Health, University of South Carolina, Columbia, SC USA; 3 Department of Epidemiology and Biostatistics, Arnold School of Public Health, University of South Carolina, Columbia, SC USA; 4 Department of Health Promotion, Education, and Behavior, Arnold School of Public Health, University of South Carolina, Columbia, SC USA

**Keywords:** Pregnancy, Maternal health, Health behaviors, Behavior change, Physical activity, Nutrition, Diet quality

## Abstract

**Background:**

Physical activity (PA), diet, and health-related quality of life (HRQOL) are related to maternal and infant health, but interventions to improve these outcomes are needed in diverse pregnant women with elevated weight.

**Methods:**

Health In Pregnancy and Postpartum (HIPP) was a randomized controlled trial. Women who were pregnant (*N*=219, 44% African American, 56% white) with overweight or obesity but otherwise healthy were randomized to a behavioral intervention grounded in Social Cognitive Theory (*n*=112) or to standard care (*n*=107). The intervention group received an in-depth counseling session, a private Facebook group, and 10 content-based counseling calls with accompanying behavioral podcasts followed by weekly or biweekly counseling calls until delivery. The standard care group received monthly mailings and 10 podcasts focused on healthy pregnancy. PA (SenseWear armband), diet (ASA24), and HRQOL (SF-12) measures were obtained from blinded assessors at baseline (<16 weeks) and late pregnancy (32 weeks). Mixed model repeated measures regression models tested treatment (Group x Time) and within-group effects. We hypothesized that intervention participants would have higher levels of PA, a better-quality diet, and higher HRQOL than standard care participants. Exploratory analyses examined whether changes in outcomes over time differed according to whether participants had recommended, excessive, or inadequate weight gain.

**Results:**

Treatment effects favored intervention participants for vegetable intake (*d*=0.40, *p*<0.05) and % whole grains (d=0.60, p<0.01). HRQOL mental component improved in both groups, but less in intervention than standard care participants (*d*=-0.33, *p*<0.05). Time effects demonstrated that total PA, steps/day, and HRQOL physical component declined significantly in both groups. Within-group effects showed that diet quality significantly improved in intervention participants. Moderate-intensity PA declined significantly in standard care participants, whereas light-intensity PA declined and sedentary behavior increased significantly in intervention participants. Finally, exploratory analyses showed that total PA and light PA increased whereas sedentary behavior decreased among those meeting guidelines for weight gain, with opposite patterns seen among those with excessive or inadequate weight gain.

**Conclusions:**

The intervention improved several dietary outcomes but had modest impacts on PA and HRQOL, underscoring the challenge of behavior change during pregnancy.

**Trial registration:**

This trial was registered in ClinicalTrials.gov on 10/09/2014. NCT02260518

**Supplementary Information:**

The online version contains supplementary material available at 10.1186/s12966-022-01387-w.

## Background

Pregnancy is a critical life period when lifestyle behaviors impact both maternal and child health outcomes. Professional organizations [1–4] emphasize the importance of healthy nutrition, regular physical activity (PA), and healthy weight gain during pregnancy. Women who enter pregnancy overweight or obese are at increased risk for deleterious health outcomes [4], and promoting healthy lifestyle practices in all women, regardless of weight status, is recommended [1].

PA during pregnancy is safe for most women and is associated with a decreased risk of deleterious birth outcomes [5] and postpartum depression [6] as well as improved health-related quality of life (HRQOL) [7]. Yet pregnant women face unique barriers to PA [8], are less active than non-pregnant women, and have a decline in PA over the course of pregnancy [9]. A recent review of PA interventions for pregnant women with elevated weight concluded that interventions are “to some extent effective” at increasing PA, although authors noted the high risk of bias in these studies, the limited use of objective PA measures, and the lack of clarity regarding theory used to guide interventions [10]. Furthermore, few studies have used objective measures to examine lower intensity PA or sedentary behavior that might be more conducive to change.

A varied, balanced, and high-quality diet is important for adequate nutrition during pregnancy, and poor health outcomes result from both undernutrition and overnutrition [4]. While some evidence indicates that pregnant women do not adhere to key dietary guidelines including vegetable and fat intake [11, 12], especially among those with lower socioeconomic status, there is also evidence suggesting that dietary intake may improve during this time [13]. Furthermore, dietary interventions have been shown to improve maternal and infant health outcomes [14] and improve dietary behaviors including increased fruit and vegetable consumption and reduced fat intake [15]. The evidence supporting the efficacy of nutrition interventions among women who are overweight or obese is less compelling due to fewer studies [16].

HRQOL is an important yet understudied outcome. More favorable HRQOL is associated with better pregnancy outcomes, but HRQOL is lower in pregnant than non-pregnant women, and the physical component of HRQOL decreases over the course of pregnancy [17]. A recent meta-analysis of intervention studies found that combined aerobic and resistance exercise, group exercise, and “yoga or PA” improved HRQOL in pregnant women, although the number of studies in each category was small [7]. A recent study found no effect of a gestational weight gain intervention on HRQOL, although higher gestational weight gain was associated with lower physical HRQOL and worsened mood across pregnancy [18].

The Health In Pregnancy and Postpartum (HIPP) trial examined a theory-based behavioral intervention versus standard care on gestational weight gain (primary outcome) as well as PA, diet, and HRQOL (secondary outcomes) among African American and white women who entered pregnancy with overweight or obesity [19]. We reported that weight gain treatment effects were moderated by race and prepregnancy weight; African American women with overweight in the behavioral intervention had significantly less gestational weight gain than their counterparts in the standard care group, with results in the opposite direction for African American women with obesity [20]. The intervention did not significantly improve moderate- to vigorous-intensity PA (MVPA) or energy intake, but these behavioral outcomes represented a very small number of the secondary behavioral outcomes that were assessed. It may have been challenging for participants with elevated weight to increase MVPA, but more realistic to increase light-intensity PA (LPA) and steps or reduce sedentary behavior. Furthermore, dietary factors beyond energy intake are important for a healthy pregnancy [21]. Thus, the purpose of this paper was to examine the impact of the behavioral intervention on objectively measured total, light-, and moderate-intensity PA (MPA), sedentary behavior, and steps; the dietary outcomes of fruit, vegetable, whole grain, sugar, and fat intake as well as diet quality; and the physical and emotional components of HRQOL. We hypothesized greater improvements (or, in the case of PA, less reduction) in all outcomes among intervention versus standard care participants. We also assessed whether these outcomes differed by race and weight status. Finally, we conducted post-hoc exploratory analyses to examine whether changes in outcomes over time differed according to whether participants had recommended, excessive, or inadequate weight gain, regardless of intervention group assignment. These categories were based on Institute of Medicine Guidelines (now National Academy of Medicine) [3].

## Methods

### Study design and participants

The CONSORT and TiDieR Checklists are included in Additional files 1 and 2. Participants were recruited primarily through 13 obstetrics and gynecology clinics in South Carolina [22]. The Institutional Review Boards from three participating healthcare centers and one university approved the study protocol. All participants signed a written informed consent form at study entry. This paper used pre-randomization baseline data collected during early (<16 weeks gestation; February 2015-January 2019) and late pregnancy (32 weeks gestation; July 2015-June 2019), described in detail elsewhere [19]. Measurement staff were blind to study assignment, and most measurement visits were conducted at the university, with home visits provided where needed. Eligibility inclusion criteria were 18-44 years of age, white or Black/African American, able to read and speak English, no plan to move from area in next 18 months, ≤16 weeks gestation, pre-pregnancy body mass index ≥25 kg/m^2^, pre-pregnancy weight ≤370 pounds (scale limitation), regular access to a telephone, and willingness to participate in weekly calls. Exclusion criteria were uncontrolled blood pressure (>160 systolic or >100 diastolic), use of insulin, uncontrolled or untreated thyroid disease, hospitalization for a mental health or substance abuse disorder in past 6 months, multiple gestation, persistent bleeding in the first trimester, physical disabilities that prevent exercise, physician advice to not exercise during pregnancy, and history of >3 miscarriages, eating disorder or malnutrition, or incompetent cervix. The sample size was determined from a power analysis that indicated 400 participants were needed to detect a small (d=0.28) intervention effects for the primary outcome (gestational weight gain) [19]. Due to recruitment challenges [22], we did not meet our recruitment goal.

### Randomization

We used a stratified randomization procedure with blocking by delivery hospital site and racial/ethnic group. Within each of the resulting eight stratums (i.e., four delivery sites x two racial/ethnic groups), for every four participants, two were randomized to the behavioral intervention group and two to the standard care group (allocation ratio = 1:1). A randomization list was generated by the statistician. The study coordinator randomized participants and forwarded the group assignment to intervention staff.

### Behavioral intervention

The behavioral intervention, described in detail elsewhere [19], was guided by Social Cognitive Theory [23] and focused on improving diet, increasing PA, and gaining healthy gestational weight. It was delivered by master’s level staff with training in public health and behavior change. All received training by the study PI (SW) in how to deliver the intervention, and they followed semi-structured scripts. During pregnancy, intervention participants received an in-depth counseling session followed by brief telephone counseling, behavioral podcasts, and access to a private Facebook group. The in-depth counseling session occurred within the first 18 weeks of gestation and provided participants feedback regarding their dietary intake and physical activity, based on their baseline dietary recalls and armband data described below, along with guidance regarding weight gain, physical activity, and dietary recommendations during pregnancy. Based on this feedback and recommendations during pregnancy, participants set initial dietary and physical activity goals and received a binder of study materials, a personalized chart to plot gestational weight gain (with upper and lower recommended bounds), a pedometer, and a bathroom scale. The weight gain chart, scale, and pedometer were used to promote self-monitoring and aid goal setting. Most sessions were conducted in person at the university, but telephone and home options were provided as needed. At the end of the session, participants were encouraged to join the study’s private Facebook group. Information about physical activity, healthy eating, and pregnancy was posted every weekday in the Facebook group to reinforce intervention content.

Participants then received 10 weekly counseling calls and 10 corresponding behavioral podcasts. The calls and podcasts focused on behavioral strategies as well as physical activity and dietary topics (see Table 1). Calls began with an assessment of changes to health. Participants were then asked to plot their weight on the chart provided in the initial counseling session. The counselor asked about progress toward the goal set on the last call and engaged the participant in problem solving to overcome barriers as needed. The new topic was then covered (behavioral strategies, physical activity, and/or diet). Finally, calls concluded with goal setting for the upcoming week, and participants were encouraged to track their physical activity and/or dietary goals on provided logs (i.e., self-monitoring). Self-regulatory behavioral strategies consistent with Social Cognitive Theory that were included in every call included self-monitoring, goal setting, and problem-solving. Further, to foster self-efficacy, participants were encouraged to set goals that were attainable. Other behavioral strategies (e.g., outcome expectations, social support, coping, stress management, time management, relapse prevention) were taught to participants via worksheets and discussions where they were encouraged to apply the strategy to their circumstances. For example, for social support, participants were asked to identify people who influenced their physical activity and dietary behaviors and discuss ways they could ask for needed support.

**Table 1:** Topics covered in the first ten pregnancy counseling calls

Call	General content	Behavioral strategy	Diet topic	Physical activity topic
1	• Study overview • Call overview	• Benefits (outcome expectations)	• Healthy eating • Benefits of healthy eating • Rate Your Plate	• Exercise safety • Benefits of exercise
2		• Goal setting • Self-monitoring	• Increasing fruits and veggies	• Self-monitoring (pedometer)
3		• Problem solving	• Myths and realities • Increasing whole grains	
4	• Breastfeeding			
5		• Enlisting social support	• Meal planning • Choosing healthy proteins/fats	
6		• Coping with emotions	• Shopping on a budget • Grocery shopping with families	• Exercise intensity
7		• Stress management		• Yoga
8		• Time management	• Reading labels • Increasing low fat forms of calcium/vitamin D	
9		• Relapse prevention	• Eating out • Fast food tips	
10	• Review of skills and topics that were most useful	• Setting postpartum goals • Postpartum planning and relapse prevention	• Healthy snacking and beverages	• Exercise options with baby • Keeping exercise fun

A similar table was included in Wilcox et al. [19] Note that self-monitoring, goal setting, problem solving, and self-efficacy were incorporated into every call

Initially, the intervention included 10 weekly group sessions that began immediately following the in-person counseling session. However, due to the challenges in recruiting an adequate number of women at one time to form a group and the less-than-ideal attendance at sessions, a protocol change was made very early in the study to replace these 10 group sessions with 10 individual telephone counseling calls (described above), allowing for rolling recruitment. All content from the group sessions was retained in the telephone counseling sessions; just the mode of delivery changed. Only one intervention group was conducted (n = 6), and these participants were retained in analyses.

When these 10 weekly calls ended, participants received briefer counseling calls until they delivered. They could opt for weekly or every-other-week calls. The calls followed the same structure as the initial 10 weekly calls (self-monitoring, goal setting, and problem solving); they did not introduce new content areas, but rather, reinforced content and skills previously covered.

### Standard Care

Standard care participants attended regularly scheduled clinic visits with their prenatal care providers. We sent participants 6 monthly mailings and links to 10 podcasts (timed consistent with the intervention group’s podcasts). These resources were commercially available and focused on having a healthy pregnancy and fetal development but did not cover PA, diet, or weight gain.

### Measures

Physical activity. The SenseWear Armband contains a 2-axis accelerometer and four sensors. It has been used in a variety of populations with most estimates of validity based on energy expenditure. It has been used as a criterion measure for PA in pregnancy [24]. Although the armband has been shown to overestimate energy expenditure in pregnancy (9% in one study and 22% in another study), it is highly related to gold standards of portable oxygen analyzer (ICC = 0.85) and indirect calorimetry (mean r = 0.93) [25, 26]. At both visits, participants were asked to wear the device for the next 8 days, with reminder and check-in calls on the 2^nd^ and 5^th^ days, and return them by mail in pre-paid envelopes. If participants did not meet the wear criteria (≥ 5 days, ≥ 1 weekend day, ≥ 21 hours/day), or if there was equipment failure, they were given the opportunity to rewear the monitor. The proprietary algorithms classify intensity of activity by metabolic equivalents (METS). For this study, minutes/day spent in total PA (>1.5 METS), LPA (1.6 to 2.9 METS), MPA (≥ 3 METS), and sedentary behavior (≤1.5 METS) were used, along with total steps/day, as continuous outcomes. MVPA was reported in a prior paper [20].

Dietary intake. Each participant completed two unannounced dietary recalls using the validated Automated Self-Administered 24-h dietary recall (ASA24) [27, 28]. One recall was conducted for a weekday and one for a weekend (Friday, Saturday, or Sunday). The first dietary recall was completed at the measurement visit and included a brief training. The second recall was scheduled within the next seven days and done on the participant’s own based on a request from study staff (randomly selected day). If the participant could not be reached, another randomly selected day was chosen. Diet quality was examined by scoring the two dietary recalls with the National Cancer Institute’s Healthy Eating Index-2015 (HEI-2015) algorithm [29]. The HEI-2015 includes 13 components that determine diet quality relative to the 2015-2019 Dietary Guidelines for Americans [30, 31]. Nine are adequacy components (e.g., total vegetables) that need to be increased, whereas four are moderation components (e.g., refined grains) that need to be reduced. The component scores are summed to create a total score with a maximum of 100 points. Krebs-Smith and colleagues [30] suggest that scores of 90-100 be graded as A, 80-89 as B, 70-79 as C, 60-69 as D, and 0-59 as F. We also reported changes in dietary intake for outcomes that were emphasized in the intervention: fruit and vegetable intake (cups/day), % of grains that were whole grains, % energy from added sugar, and % energy from saturated fat. We did not examine outcomes that were discussed with participants largely within the context of reducing saturated fat, such as meat and dairy, because cheese, beef, and dairy are the top food sources of saturated fat [32]. Energy intake (kcal/day) was reported in a prior paper [20].

Health-related quality of life. The 12-item Short Form (SF-12) measured HRQOL [33]. This widely-used measure, including during pregnancy [17], assesses eight areas over the past four weeks: physical functioning, role physical, bodily pain, general health perceptions, vitality, social functioning, role emotional, and mental health. Items are summed and yield a physical and a mental component summary scale. Higher scores indicate more favorable HRQOL.

Demographic and pregnancy-related variables. Participants provided extensive demographic and pregnancy-related information at baseline. Several of these variables were included in our statistical models because of their potential relationships with PA, diet, and/or HRQOL: maternal age, baseline gestational weeks, race (white or Black/African American), parity (nulliparous vs. not nulliparous), pre-pregnancy weight status (overweight vs. obese, based on measured height and self-reported weight), and education (college education vs. lower level of education).

### Analyses

We conducted intent-to-treat repeated measures analyses using SAS version 9.4. To use all available data and reduce bias in estimates due to missing data at 32 weeks [34], we used multiple linear mixed models (PROC MIXED) that adjusted for demographic and pregnancy-related variables. Treatment effects were tested with Group x Time interactions. For several models, analyses were re-run using a square root transformation of the outcome variable because of the potential for normality of residuals violations. The transformations corrected violations, but significance levels were not impacted, suggesting the models were robust in the presence of non-normality. Thus, we retained the models with their original outcome variables for simplicity in interpreting findings. We also examined whether treatment effects differed across categories of pre-pregnancy weight status and race by including three-way (Group x Time x Weight status and Group x Time x Race) and four-way (Group x Time x Weight status x Race) interaction terms. Because no higher order interactions were significant, nor did they improve model fit substantially, we only reported models with the Group x Time interaction. We computed intervention effect sizes (d) to show the magnitude of treatment effects. In models where the Group x Time interaction was not significant, we also examined whether there were within-group effects (differences by time within each of the two groups). For each models, we reported whether any of the health-related or demographic variables (main effects) were associated with the outcome.

Lastly, we conducted exploratory analyses to examine whether those who met the IOM guidelines were more likely than those who had excessive or inadequate weight gain to show improvements on PA, diet, and HRQOL outcomes. The analyses examined IOM Group x Time interactions, and retained the same covariates described above.

## Results

### Sample

**Fig. 1:**
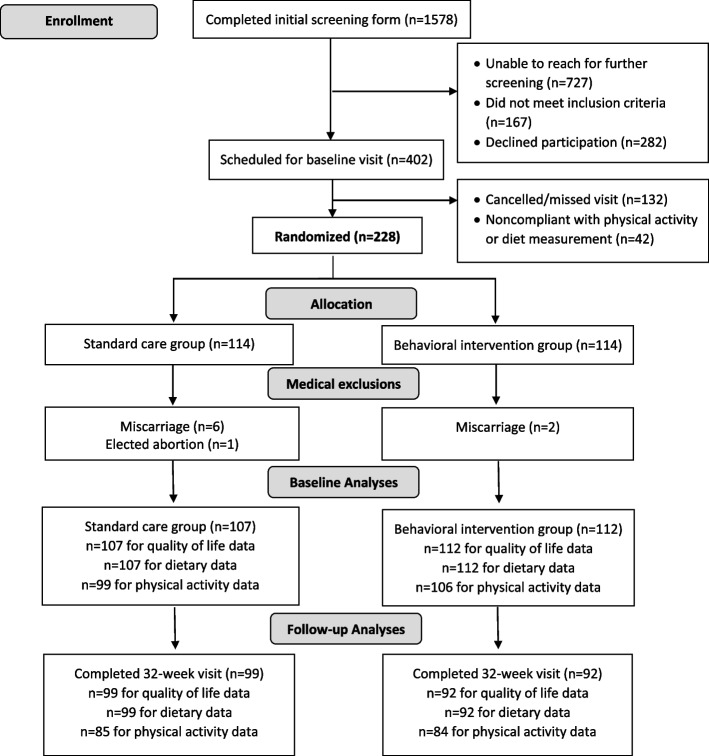
Recruitment and retention of study participants

As shown in Fig. 1, 228 participants were randomized. Nine were withdrawn by research staff due to becoming ineligible, resulting in a sample of 219 (112 intervention, 107 standard care). Baseline dietary and HRQOL data were available for all participants. The second baseline dietary recall was completed, on average, 4.4 ± 2.6 days after the first dietary recall (range: 1 to 17 days). Thirteen women at baseline reported nickel allergies and were unable to wear the SenseWear armband, and data could not be located for an additional participant. At 32 weeks, 191 participants (87%) completed the measurement visit; all completed the dietary and HRQOL measures, but 22 did not wear the SenseWear armband. Study adverse events during pregnancy (*n*=24; 11 behavioral intervention, 13 standard care) are reported elsewhere [35], with all determined to be unrelated to the intervention.

The baseline demographic, PA, dietary, and HRQOL variables are reported in Table 2. Nearly half of sample participants were African American (44.3%) and nulliparous (42.9%), and just over half were college graduates (59.4%). Over half were married (67.1%) and employed full-time (61.2%). Participants averaged 30 years of age and, on average, were at the end of the first trimester of their pregnancy at baseline. Participants were nearly evenly split between overweight and obese weight categories.

**Table 2:** Baseline characteristics of study participants (*N*=219), by randomization assignment

	Behavioral Intervention (*n*=112)		Standard Care (*n*=107)	
Characteristic	Mean (SD)	% (n)	Mean (SD)	% (n)
Race, %				
Black/African American		42.0 (47)		46.7 (50)
White		58.0 (65)		53.3 (57)
Married, %		75.0 (84)		58.9 (63)
College graduate, %		59.2 (67)		58.9 (63)
Household income, %				
<$35,000		22.3 (25)		35.5 (38)
$35,000-$49,999		15.2 (17)		12.1 (13)
$50,000-$74,999		17.9 (20)		20.6 (22)
≥$75,000		42.9 (48)		31.8 (34)
Employed full time, %		61.6 (69)		60.8 (65)
Nulliparous, %		43.8 (49)		42.1 (45)
Age, years	30.4 (5.2)		29.1 (4.8)	
Gestation at baseline, weeks	12.6 (2.4)		12.6 (2.3)	
Pre-pregnancy BMI, kg/m^2^	33.0 (6.6)		33.9 (6.1)	
Overweight, %		50.0 (56)		53.3 (57)
Obese, %		50.0 (56)		46.7 (50)
Armband wear time, minutes/day	1404.9 (24.0)		1404.6 (24.6)	
Armband, total compliant days	6.9 (1.1)		7.0 (0.8)	
Total PA, minutes/day	256.2 (95.4)		247.9 (99.8)	
Light PA, minutes/day	218.2 (83.5)		200.1 (77.1)	
MPA, minutes/day	37.6 (21.2)		34.8 (22.8)	
VPA, minutes/day	0.4 (1.0)		0.3 (1.5)	
MVPA, minutes/day	38.0 (21.4)		35.2 (23.4)	
Sedentary minutes/day	1148.6 (98.5)		1169.4 (95.0)	
Steps/day	5560.7 (2021.5)		5145.1 (2296.6)	
Diet quality (HEI-2015)	53.1 (13.0)		50.9 (10.4)	
Kcals/day	1857.0 (489.6)		2013.5 (729.5)	
Fruit, cup equivalents/day	1.2 (1.1)		1.0 (1.2)	
Vegetables, cup equivalents/day	1.6 (1.0)		1.7 (1.2)	
Whole grains, ounce equivalents/day	0.7 (0.8)		0.7 (0.9)	
% Whole grains	12.4 (14.6)		11.5 (14.2)	
Added sugar, % of kcals	12.3 (8.2)		11.4 (7.0)	
Total fat, % of kcals	36.4 (6.8)		37.2 (7.0)	
Saturated fat, % of kcals	11.7 (2.9)		12.6 (2.9)	
HRQOL – mental component	51.0 (7.6)		49.6 (6.8)	
HRQOL – physical component	47.7 (7.5)		47.6 (6.8)	

*Kg* kilogram, *M* meters, *BMI* body mass index, *PA* physical activity, *LPA* light-intensity physical activity, *MPA* moderate-intensity physical activity, *VPA* vigorous-intensity physical activity, *MVPA* moderate- to vigorous-intensity physical activity, *Kcals* kilocalories *HEI-2015* Healthy Eating Index 2015, *HRQOL* health-related quality of life, *SD* standard deviation

### Main outcomes – treatment effects for PA, diet, and HRQOL

As shown in Table 3, there were no significant treatment effects (Group x Time) for any of the PA outcomes - total PA, LPA, MPA, sedentary behavior, or steps. Treatment effects favoring the intervention group were statistically significant for vegetables and % of grains that were whole grains. Standard care participants significantly reduced vegetable consumption, whereas intervention participants significantly increased the % of grains that were whole grains. Effect sizes for these differences approached or were in the moderate range (d = 0.40 and d = 0.60). Finally, treatment effects were statistically significant for the mental but not physical component of HRQOL. While scores on the mental component significantly improved from early to late pregnancy for both groups, the increase was greater in the standard care group. The treatment effect size was small in magnitude (d = -0.31).

**Table 3:** Results of multiple mixed model regression analyses testing intervention effects for physical activity, diet, and health-related quality of life outcomes

		Behavioral Intervention LSMs			Standard Care LSMs			Group x Time		
Dependent Variable	Recs	Baseline	Late Pregnancy	Within Grp Diff	Baseline	Late Pregnancy	Within Grp Diff	Between Grp Diff	d	p
Total PA min/day	n/a	249.12	233.85	-15.27*	229.72	216.50	-13.22*	-2.05	-0.02	0.82
Light PA min/day	n/a	212.32	200.02	-12.30*	194.10	188.85	-5.25	-7.05	-0.09	0.42
Moderate PA min/day	n/a	36.51	34.08	-2.43	35.24	27.95	-7.29*	4.86	0.22	0.17
Sedentary min/day	n/a	1155.12	1170.06	14.94*	1175.14	1185.10	9.96	4.98	0.05	0.62
Steps/day	n/a	5375.08	4838.31	-536.77*	5002.12	4220.24	-781.88*	245.11	0.11	0.29
Diet quality (HEI-2015)	n/a	52.71	55.98	3.27*	50.78	52.71	1.93	1.34	0.11	0.51
Fruit, cup equiv/day	2.0	1.19	1.44	0.26	1.02	1.23	0.21	0.05	0.05	0.84
Vegetables, cup equiv/day	2.5	1.58	1.74	0.16	1.73	1.44	-0.29*	0.44	0.40	**0.01**
% whole grains	50	11.81	19.86	8.05*	11.61	11.06	-0.55	8.59	0.60	**<0.01**
Added sugar, % of kcals	10	12.22	11.54	-0.68	11.15	11.13	-0.02	-0.70	-0.09	0.54
Saturated fat, % of kcals	10	11.65	11.94	0.28	12.72	12.03	-0.69	0.97	0.33	0.08
HRQOL – mental component	n/a	50.81	52.65	1.84*	49.58	54.10	4.52*	-2.68	-0.31	**0.03**
HRQOL – physical component	n/a	47.45	41.33	-6.12*	47.15	39.06	-8.09*	1.97	0.28	0.15

* *p*<.05 for within-group changes over time

Recs = Recommended value based on Dietary Guidelines for Americans, 2020-2025, based on a 2,000 kcal/d diet

d = Effect size d = [(intervention 32 weeks LSM – intervention baseline LSM) – (standard care 32 weeks LSM – standard care baseline LSM)]/pooled and unadjusted baseline standard deviation. Effect sizes of d = |0.20| were considered small, d = |0.50| medium, and d = |0.80| large

*LSM* least square means, *PA* physical activity, *Equiv* equivalents, *HRQOL* health-related quality of life

The mixed multiple regression models (and LSMs) were adjusted for maternal age, gestational age at baseline, race (African American vs. white), parity (nulliparous vs. not), pre-pregnancy weight status (overweight vs obese), and educational attainment (college graduate vs. less education)

Note: Moderate- to vigorous-intensity physical activity and energy intake (kcals/day) were reported in an earlier paper [20] and thus are not included in this paper

### Secondary outcomes – within-group changes for PA, diet, and HRQOL

Within-group changes over time for PA are shown in Additional files 3 and 4. Total PA mins/day and steps/day decreased significantly in both groups, but the declines were somewhat greater for standard care participants. MPA decreased only in the standard care group, whereas LPA decreased only in the intervention group. Finally, sedentary behavior increased significantly only in intervention participants. Within-group changes over time for diet are shown in Additional files 5 and 6). Beyond the treatment effects reported earlier for vegetable intake and % of grains that were whole grains, we also found significant improvements in diet quality among intervention participants. Within-group changes were not seen for fruit, sugar, or saturated fat. Within-group changes over time for HRQOL are shown in Additional file 7. Beyond the treatment effects reported earlier for mental HRQOL, both groups reported a reduction in the physical component over time, but this reduction was somewhat smaller in intervention women.

### Secondary outcomes - health-related and sociodemographic main effects

Several main effects were also statistically significant for PA (Additional files 3 and 4). African American women were less active (total PA, LPA, MPA, steps) and more sedentary than white women. Women with overweight were more active (total PA, LPA, MPA) and less sedentary than women with obesity. Women with at least one live birth (i.e., not nulliparous) had more total PA and LPA and less sedentary time than nulliparous women. Age was negatively associated with LPA. Finally, college graduates had more steps/day than those with less education. Baseline gestational weeks were unrelated to PA variables.

For diet (Additional files 5 and 6), participants with higher education had higher dietary quality scores. Age was positively associated with vegetable consumption, % of grains that were whole grains, and % energy from total fat, and negatively associated with % energy from added sugars. Finally, African American participants had a lower % energy from sugar than white participants. The main effects of weight status, parity, and baseline gestational weeks were unrelated to dietary variables.

For HRQOL (Additional file 7), the physical component scores were lower in African American than white participants, negatively associated with age, and higher in participants with overweight (vs. obesity) and a college education (vs. lower level of education) participants.

### Exploratory analyses – differences in outcomes according to meeting weight gain guidelines

As shown in Table 4, the IOM Group x Time interactions were significant for total PA, light PA, and sedentary behavior, and approached significance for MPA, such that those meeting guidelines had significantly more favorable changes over time than those with excessive or inadequate weight gain. For total PA, those with excessive (*p*<.001) and inadequate (*p*<.01) weight gain differed significantly over time from those meeting guidelines. Those meeting guidelines increased total PA and the other two groups reduced total PA. For LPA, the same group comparisons were significant (*p*<.01 for excessive, *p*<.01 for inadequate) and the same pattern emerged. For sedentary activity, the same group comparisons were significant (*p*<.01 for excessive, *p*<.01 for inadequate), but those meeting guidelines decreased sedentary time while the other two groups increased sedentary time. Finally, for MPA, those exceeding guidelines had different patterns over time (decreased MPA) as compared to those who met guidelines (increased MPA; *p*<.02). The IOM Group x Time interaction was not significant for steps (*p*=.11), although steps appeared to decline less for those meeting guidelines as compared to the other two groups. No IOM Group x Time interactions were found for any of the diet or HRQOL variables (data not shown).

**Table 4:** Results of multiple mixed model regression analyses testing whether those who met IOM guidelines had increased physical activity outcomes relative to those with excessive and inadequate weight gain

	Recommended Weight Gain LSMs		Excessive Weight Gain LSMs		Inadequate Weight Gain LSMs		IOM x Time
Dependent Variable	Baseline	Late Pregnancy	Baseline	Late Pregnancy	Baseline	Late Pregnancy	p
Total PA min/day	236.18	254.86	244.59	222.23	224.73	195.50	0.04
Light PA min/day	200.46	216.34	207.98	193.38	189.98	167.58	<0.01
Moderate PA min/day	35.42	38.76	36.28	28.97	34.19	29.02	0.07
Sedentary min/day	1165.59	1149.38	1160.41	1179.51	1180.43	1208.25	<0.01
Steps/day	4792.42	4554.39	5359.65	4651.82	4987.16	3959.63	0.12

*LSM* least square means, *PA* physical activity, *IOM* Institute of Medicine (now called National Academy of Medicine)

## Discussion

This paper reported the PA, diet, and HRQOL secondary outcomes from the HIPP trial, a RCT comparing a theory-based behavioral intervention to standard care among an important but understudied group - Black/African American and white women who entered pregnancy with overweight or obesity. We hypothesized that participants in the intervention group would show improvements in these outcomes from early to late pregnancy relative to the standard care group.

Treatment effects were not found for any PA outcome. Total PA and steps decreased significantly in both groups. The pattern of findings, however, differed by group: the decrease in total PA was due to reduced LPA among intervention participants versus reduced MPA among standard care participants. Furthermore, when minutes/day of MPA (relatively high) were compared to steps/day (relatively low), our data suggest that few participants were engaging in structured or planned exercise, and their step data indicates they were underactive [36]. Although two reviews of PA interventions during pregnancy reported that interventions generally increased PA [37, 38], all but one study in these reviews used self-reported PA measures. Self-report measures are prone to overreporting biases and show only slight to fair agreement with objective measures [39]. Thus, it is possible that our study differs from the literature because we used an objective measure of PA. In the one study that used accelerometers [40], there was an 18% reduction in PA among control participants and a 25% reduction among intervention participants. A recent review of 19 PA interventions for pregnant women with overweight and obesity included 8 studies with objective measures; significant intervention effects were found in 1 of 2 studies that used accelerometers, 0 of 4 studies that used pedometers, and 3 of 3 studies that assessed VO_2_ max or heart rate [10]. Given the consistent findings that pregnant women are less active than non-pregnant women and that PA declines over the course of pregnancy [8], combined with the documented benefits of PA during pregnancy [38, 41], continued emphasis must be placed on addressing barriers to PA among pregnant women [8] and including objective PA measures. We also found that African American women, women with obesity, and nulliparous women were significantly less active and more sedentary than their counterparts, underscoring priority populations for future studies.

Our exploratory analyses showed that PA patterns from early to late pregnancy were more favorable for those meeting IOM weight gain guidelines as compared to those with excessive or inadequate weight gain, irrespective of intervention group assignment. This finding suggests that PA may play a role in fostering healthy weight gain, consistent with a recent review [14]. It is also possible that participants who were the most weight conscious were also the most likely to use PA for weight control.

The dietary intake of our study participants was concerning. Diet quality scores were lower than national norms [31], substantially lower than scores shown in other studies of pregnant women [42–45], and in the “failing” category proposed by Krebs-Smith and colleagues [30]. Mean scores in our sample were comparable, however, to another study where women with overweight/obesity had lower HEI scores than normal weight women [46]. Although we did not find a treatment effect for diet quality, it increased significantly in intervention but not standard care participants (within-subjects effect). We found significant intervention effects favoring the intervention group for two dietary outcomes that were particularly low at baseline: vegetable intake and % of grains that were whole grains. While dietary intake for these outcomes during late pregnancy remained well below recommendations, these improvements nonetheless represent meaningful changes. Zhu et al. [47] found that when pregnant women substitute just one serving of refined grain with whole grains, they have a 10% reduced risk of having a child with overweight or obesity. Contrary to hypotheses, we found no intervention effects for fruit; percentage of energy from added sugar, or saturated fat; or energy intake (kcals/day, reported in an earlier paper [20]). A recent systematic review of systematic reviews concluded there is consistent evidence that dietary interventions during pregnancy increase fruit and vegetable consumption and “fairly consistent” evidence that they reduce fat intake [15]. There was not consistent evidence for other areas of diet, and the authors noted that most reviews prioritized health-related outcomes over behavioral outcomes.

Our study also examined whether the intervention improved HRQOL, an outcome that is understudied despite evidence that HRQOL is lower in pregnant women relative to their peers, appears to decrease over the course of pregnancy (especially for the physical component), and is associated with pregnancy outcomes [17, 18]. Consistent with a systematic review [17], we found that the mental component of HRQOL increased whereas the physical component decreased from early to late pregnancy in both groups. Although the mental component improved significantly in both groups, contrary to hypotheses, the improvement was greater in standard care than intervention participants. The mean component scores in our study were similar to other pregnancy studies [18, 48] and lower than the general population. Relatively few studies have examined the impact of PA on QOL in pregnancy, and results have not yielded consistent findings [7, 18, 38]. Our study results suggest that future work should differentiate physical from mental components of QOL among pregnant women.

There are several possible explanations for why our intervention had modest to no impacts on PA, diet, and HRQOL. Because we were not successful at improving PA, it is not surprising that we found no treatment effects for the physical component of HRQOL. The mental component of HRQOL improved in both groups (significantly more so in the standard care group) even though we did not intervene on psychological factors, whereas the physical component decreased in both groups (less so in the intervention group). Therefore, the changes we saw mirrored what has been reported in pregnancy in the absence of an intervention [17]. Regarding PA and diet, it is possible that our intervention was too broad, and that it was overwhelming for women to focus on PA, diet, and healthy weight gain simultaneously. In support of this speculation, Teede et al.’s [14] recent review of interventions to promote healthy gestational weight gain found that diet only interventions led to more favorable (i.e., less) gestational weight than PA interventions, diet with PA interventions, or mixed interventions. Indeed, the challenges related to increasing PA are substantial [49] and often requiring adding in a new behavior that may be challenging and time-consuming versus making modifications to existing behaviors, as is the case for diet. Behavioral intervention participants in our study improved their diet quality, vegetable intake, and % of grains that were whole grains, suggesting they were able to make small but meaningful changes to their diet. Furthermore, in Teede et al.’s review, diet interventions and PA interventions had favorable impacts on more adverse pregnancy outcomes than did diet with PA interventions and mixed interventions. It is possible that narrowing the targets of the intervention (diet-only or PA-only) or focusing on the goal behaviors in a sequential manner (e.g., add PA once dietary changes are made), might yield greater behavior change than focusing on all behaviors simultaneously. Our lack of findings could also relate to the characteristics of our study sample, which was ethnically diverse and resided in the southern United States. Finally, it is possible that behavioral intervention participants did not receive an adequate dose of the intervention. Although we have reported in a previous paper that call completion and podcast downloads were high during pregnancy in the intervention group, we also found that completion of the initial counseling session and the number of the initial 10 calls received related significantly and favorably to gestational weight gain (i.e., more intervention content, less weight gain) [50]. Research is needed to help disentangle how best to help pregnant women make important lifestyle changes.

There are several study limitations. First, we did not meet our recruitment goal of 400 participants despite recruiting from 13 clinics over a 4-year period. We reported recruitment challenges in a previous paper [22]. Others have reported their challenges in recruiting pregnant women. For example, in the LIFE-Moms trials [51], a consortium of seven independent but collaborative clinical trials focused on excessive gestational weight in women with overweight or obesity, recruitment at three of the seven sites was stopped early by the funder as they were deemed unlikely to reach their target recruitment goal over the three-year recruitment period. These sites enrolled only 31/200, 54/200, and 43/306 participants (sample sizes in the other seven ranged from 205 to 280). Although our sample was larger than over half of the existing diet and PA interventions in recent reviews [10, 16, 37, 38], we likely did not have adequate statistical power to detect higher order interactions (e.g., treatment moderators). Also, although effect sizes for our secondary outcomes that were in the small to moderate range (e.g., d=0.31 to 0.60) were statistically significant, effect sizes for the smaller treatment effects of minutes/week of MPA (d=0.22), % saturated fat (d=0.33), and HRQOL physical component (d=0.28) only approached statistical significance. Our inclusion of effect sizes provides useful information to judge the clinical meaningfulness of outcomes. Second, recruitment from one region of one state might limit generalizability to other areas of the U.S. and beyond. Third, even though we equated the number of podcasts across study groups, intervention participants received more attention than standard care participants. Fourth, we cannot rule out social desirability biases for our dietary findings, although improvements were not seen across all dietary outcomes.

A major study strength is that we used an objective measure of PA. Other strengths include that we recruited a sample that is large relative to other intervention studies during pregnancy, nearly half of the sample were Black/African American women, all participants were overweight or obese, and we tested a comprehensive, theory-based intervention.

## Conclusions

Overall, this study highlights that pregnancy is a challenging time to promote health behavior changes among women with elevated weight. While we observed some improvements in diet, PA and HRQOL were harder to impact. Future research might benefit from adaptive or stepped care designs that add more intensive intervention components to participants who do not reach behavioral goals and thus use resources more wisely. There is also a need to better understand the timing of when and in what order to introduce PA and diet content, which might be influenced by the preferences of participants. Helping pregnant women integrate behavioral changes into their lives is important for enhancing both maternal and infant health outcomes.

## Supplementary Information

**Additional file 1.**

**Additional file 2.**

**Additional file 3.**

## Data Availability

Data are not included in a repository but are available from the corresponding author on reasonable request.

## Abbreviations

ASA24: Automated Self-Administered 24-h dietary recall
HEI-2015: Healthy Eating Index-2015
HRQOL: health-related quality of life
LPA: light-intensity physical activity
MET: metabolic equivalent
MPA: moderate-intensity physical activity
MVPA: moderate- to vigorous-intensity physical activity
PA: physical activity
