# Supplementary material for: Effects of a behavioral intervention on physical activity, diet, and health-related quality of life in pregnant women with elevated weight: results of the HIPP randomized controlled trial
Source: Int J Behav Nutr Phys Act. 2022 Dec 9;19:145. doi: 10.1186/s12966-022-01387-w (PMC9733107; doi:10.1186/s12966-022-01387-w)
Supplement: Supplementary file 3 — Additional file 3. [file 12966_2022_1387_MOESM3_ESM.docx]

Additional File 3. Full Mixed Linear Regression Models – Total Physical Activity, Light-Intensity Physical Activity, and Moderate-Intensity Physical Activity Outcomes

|  | Total PA mins/day | | |  | LPA mins/day | | |  | MPA | | |
| --- | --- | --- | --- | --- | --- | --- | --- | --- | --- | --- | --- |
|  | B | F | p |  | B | F | p |  | B | F | p |
| Group (intervention) | 19.48 | 2.57 | 0.11 |  | 18.22 | 2.34 | 0.13 |  | 1.27 | 1.69 | 0.20 |
| Time | -13.21 | 9.24 | **<0.01** |  | -5.24 | 4.15 | **<0.05** |  | -7.28 | 7.64 | **<0.01** |
| Group x Time | -2.13 | 0.05 | 0.82 |  | -7.05 | 0.67 | 0.41 |  | 4.85 | 1.90 | 0.17 |
| Time within intervention group | -15.34 | 5.38 | **<0.05** |  | -12.29 | 4.11 | **<0.05** |  | -2.44 | 0.97 | 0.33 |
| Time within standard care group | -13.21 | 3.93 | **<0.05** |  | -5.24 | 0.74 | 0.39 |  | -7.28 | 8.50 | **<0.01** |
| Race (African American) | -39.47 | 10.99 | **<0.01** |  | -29.40 | 8.69 | **<0.01** |  | -10.33 | 12.25 | **<0.001** |
| Weight status (overweight) | 63.94 | 30.32 | **<0.001** |  | 49.23 | 25.65 | **<0.0001** |  | 14.18 | 24.37 | **<0.0001** |
| Age (years) | -2.17 | 2.83 | 0.09 |  | -2.49 | 5.30 | **<0.05** |  | 0.32 | 0.99 | 0.32 |
| Parity (not nulliparous) | 52.13 | 18.06 | **<0.0001** |  | 51.75 | 25.39 | **<0.0001** |  | 0.24 | 0.01 | 0.94 |
| Education (college graduate) | 15.95 | 1.64 | 0.20 |  | 13.76 | 1.74 | 0.19 |  | 1.89 | 0.38 | 0.54 |
| Gestation (weeks) | -0.11 | 0.00 | 0.96 |  | 0.68 | 0.11 | 0.74 |  | -0.74 | 1.45 | 0.23 |

PA = physical activity. Mins = Minutes. MPA = moderate-intensity physical activity. LPA = light-intensity physical activity.

Note: These results are from a multiple mixed linear regression model (PROC MIXED) and estimates (B) are adjusted for all other variables in the model. A previous paper reported results for moderate- to vigorous-intensity physical activity.

Additional File 4. Full Mixed Linear Regression Models – Sedentary Behavioral and Steps Outcomes

|  | Sedentary mins/d | | |  | Steps/day | | |
| --- | --- | --- | --- | --- | --- | --- | --- |
|  | B | F | p |  | B | F | p |
| Group (intervention) | -20.02 | 2.14 | 0.15 |  | 372.96 | 3.94 | **<0.05** |
| Time | 9.96 | 6.28 | **<0.05** |  | -781.89 | 32.82 | **<0.0001** |
| Group x Time | 4.99 | 0.25 | 0.62 |  | 245.11 | 1.13 | 0.29 |
| Time within intervention group | 14.95 | 4.55 | **<0.05** |  | -536.77 | 10.96 | **<0.01** |
| Time within standard care group | 9.96 | 1.99 | 0.16 |  | -781.89 | 22.90 | **<0.0001** |
| Race (African American) | 31.55 | 6.45 | **<0.05** |  | -1199.20 | 21.42 | **<0.0001** |
| Weight status (overweight) | -67.81 | 31.35 | **<0.0001** |  | 353.98 | 1.96 | 0.16 |
| Age (years) | 2.54 | 3.58 | 0.06 |  | -6.38 | 0.05 | 0.82 |
| Parity (not nulliparous) | -54.62 | 18.22 | **<0.0001** |  | 491.71 | 3.39 | 0.07 |
| Education (college graduate) | -16.81 | 1.68 | 0.20 |  | 602.70 | 4.95 | **<0.05** |
| Gestation (weeks) | 0.12 | 0.00 | 0.96 |  | -32.51 | 0.36 | 0.55 |

Mins = minutes.

Note: These results are from a multiple mixed linear regression model (PROC MIXED) and estimates (B) are adjusted for all other variables in the model.

Additional File 5. Full Mixed Linear Regression Models – Diet Quality, Fruit Consumption, and Vegetable Consumption

|  | Diet quality (HEI-2015) | | |  | Fruit cup/d | | |  | Vegetables cup/d | | |
| --- | --- | --- | --- | --- | --- | --- | --- | --- | --- | --- | --- |
|  | B | F | p |  | B | F | p |  | B | F | p |
| Group (intervention) | 1.93 | 3.89 | **0.049** |  | 0.16 | 1.27 | 0.26 |  | -0.15 | 0.31 | 0.58 |
| Time | 1.93 | 6.70 | **<0.05** |  | 0.21 | 3.65 | 0.06 |  | -0.29 | 0.57 | 0.45 |
| Group x Time | 1.34 | 0.44 | 0.51 |  | 0.05 | 0.04 | 0.84 |  | 0.44 | 6.35 | **<0.05** |
| Time within intervention group | 3.27 | 5.21 | **<0.05** |  | 0.26 | 2.2 | 0.14 |  | 0.16 | 1.53 | 0.22 |
| Time within standard care group | 1.96 | 1.88 | 0.17 |  | 0.21 | 1.48 | 0.23 |  | -0.29 | 5.46 | **<0.05** |
| Race (African American) | 0.07 | 0.00 | 0.96 |  | 0.21 | 1.43 | 0.23 |  | -0.01 | 0.00 | 0.95 |
| Weight status (overweight) | -0.69 | 0.27 | 0.60 |  | 0.08 | 0.22 | 0.64 |  | 0.03 | 0.05 | 0.83 |
| Age (years) | 0.26 | 3.01 | 0.08 |  | 0.00 | 0.01 | 0.92 |  | 0.04 | 7.24 | **<0.01** |
| Parity (not nulliparous) | -0.60 | 0.18 | 0.67 |  | 0.12 | 0.47 | 0.49 |  | -0.07 | 0.28 | 0.60 |
| Education (college graduate) | 3.01 | 4.42 | **<0.05** |  | -0.15 | .069 | 0.41 |  | 0.07 | 0.30 | 0.58 |
| Gestation (weeks) | 0.16 | 0.31 | 0.58 |  | 0.02 | 0.26 | 0.61 |  | 0.02 | 0.74 | 0.39 |

Note: These results are from a multiple mixed linear regression model (PROC MIXED) and estimates (B) are adjusted for all other variables in the model. Results for Energy intake (kcals/d) were reported in an earlier paper. HEI-2015 = Healthy Eating Index-2015.

Additional File 6. Full Mixed Linear Regression Models – Whole Grains, Added Sugar, and Saturated Fat

|  |  | % Grains that are whole grain | | |  | % energy from added sugar | | |  | % energy from saturated fat | | |
| --- | --- | --- | --- | --- | --- | --- | --- | --- | --- | --- | --- | --- |
|  |  | B | F | p |  | B | F | p |  | B | F | p |
| Group (intervention) |  | 0.002 | 6.76 | **<0.05** |  | 1.09 | 0.87 | 0.35 |  | -1.07 | 3.17 | 0.08 |
| Time |  | -0.01 | 7.08 | **<0.01** |  | 0.02 | 0.32 | 0.57 |  | -0.69 | 0.54 | 0.46 |
| Group x Time |  | 0.08 | 9.27 | **<0.01** |  | -0.70 | 0.37 | 0.54 |  | 0.97 | 3.13 | 0.08 |
| Time within intervention group |  | 0.08 | 16.04 | **<0.0001** |  | -0.68 | 0.68 | 0.41 |  | 0.28 | 0.53 | 0.47 |
| Time within standard care group |  | -0.005 | 0.08 | 0.78 |  | 0.02 | 0.00 | 0.98 |  | -0.69 | 3.17 | 0.08 |
| Race (African American) |  | -0.02 | 0.81 | 0.37 |  | -2.38 | 8.25 | **<0.01** |  | -0.32 | 0.90 | 0.34 |
| Weight status (overweight) |  | 0.004 | 0.06 | 0.81 |  | 0.82 | 1.05 | 0.31 |  | 0.16 | 0.24 | 0.62 |
| Age (years) |  | 0.004 | 4.41 | **<0.05** |  | -0.26 | 8.52 | **<0.01** |  | 0.06 | 2.76 | 0.10 |
| Parity (not nulliparous) |  | -0.02 | 1.33 | 0.25 |  | 0.55 | 0.43 | 0.51 |  | -0.36 | 1.08 | 0.30 |
| Education (college graduate) |  | 0.03 | 2.30 | 0.13 |  | -0.12 | 0.02 | 0.89 |  | -0.19 | 0.28 | 0.60 |
| Gestation (weeks) |  | 0.00 | 0.02 | 0.88 |  | -0.02 | 0.01 | 0.91 |  | -0.002 | 0.00 | 0.98 |

Note: These results are from a multiple mixed linear regression model (PROC MIXED) and estimates (B) are adjusted for all other variables in the model.

Additional File 7. Full Mixed Linear Regression Models – Physical and Mental Health-Related Quality of Life

|  | Mental | | |  | Physical | | |
| --- | --- | --- | --- | --- | --- | --- | --- |
|  | B | F | p |  | B | F | p |
| Group (intervention) | 1.23 | 0.01 | 0.91 |  | 0.30 | 2.05 | 0.15 |
| Time | 4.52 | 26.24 | **<0.0001** |  | -8.09 | 108.63 | **<0.0001** |
| Group x Time | -2.68 | 4.65 | **<0.05** |  | 1.97 | 2.09 | 0.15 |
| Time within intervention group | 1.84 | 4.30 | **<0.05** |  | -6.12 | 39.62 | **<0.0001** |
| Time within standard care group | 4.52 | 27.08 | **<0.0001** |  | -8.09 | 71.60 | **<0.0001** |
| Race (African American) | -0.23 | 0.05 | 0.82 |  | -1.96 | 4.42 | **<0.05** |
| Weight status (overweight) | 0.89 | 0.77 | 0.38 |  | 2.47 | 7.50 | **<0.01** |
| Age (years) | 0.10 | 0.74 | 0.39 |  | -0.31 | 9.83 | **<0.01** |
| Parity (not nulliparous) | 0.44 | 0.17 | 0.68 |  | 0.53 | 0.32 | 0.57 |
| Education (college graduate) | 0.60 | 0.31 | 0.58 |  | 2.75 | 7.93 | **<0.01** |
| Gestation (weeks) | -0.14 | 0.42 | 0.52 |  | -0.23 | 1.41 | 0.24 |

Note: These results are from a multiple mixed linear regression model (PROC MIXED) and estimates (B) are adjusted for all other variables in the model.
